# JAK Inhibitors and Oxidative Stress Control

**DOI:** 10.3389/fimmu.2019.02814

**Published:** 2019-12-06

**Authors:** Amandine Charras, Pinelopi Arvaniti, Christelle Le Dantec, George N. Dalekos, Kaliopi Zachou, Anne Bordron, Yves Renaudineau

**Affiliations:** ^1^UMR1227, Lymphocytes B et Autoimmunité, Université de Brest, INSERM, CHU de Brest, Brest, France; ^2^Laboratory of Immunology and Immunotherapy, Brest University Medical School Hospital, Brest, France; ^3^Institute of Internal Medicine and Hepatology, Larissa, Greece; ^4^Department of Medicine and Research Laboratory of Internal Medicine, University Hospital of Larissa, Larissa, Greece

**Keywords:** primary sjögren's syndrome, ROS, JAK/STAT pathway, ICAM-1, PD-L1

## Abstract

Primary Sjögren's syndrome (SjS) is a complex autoimmune epithelitis, with few treatment options, but the use of Janus kinase (JAK) inhibitors is promising because suppression of the JAK/signal transducer and activator of transcription (STAT) pathway improves sicca manifestations. Playing a primary and pathogenic role in disease development, the oxidative stress response is upregulated in activated salivary gland epithelial cells (SGECs) from patients with SjS. Therefore, the aim of this study was to investigate whether JAK inhibitors would suppress SGEC activation in response to an oxidative stress. For this purpose, the human salivary gland (HSG) cell line was used, and cells were treated with the reactive oxygen species (ROS) inducer hydrogen peroxide (H_2_O_2_) or with interferons (IFN Type I and Type II), used as positive controls, to mimic activated SGECs as observed in SjS patients. Afterward, the levels of the intracellular adhesion molecule-1 (ICAM-1) and the regulatory programmed-death ligand-1 (PD-L1) were measured by real-time PCR and flow cytometry, and the STAT1/3 phosphorylation status was assessed by Western blotting. Using the HSG cell line, our results showed that both ICAM-1 and PD-L1 are induced by ROS through pSTAT3, and that this activation pathway is reversed by the use of JAK inhibitors, AG490 and ruxolitinib, as well as by N-acetylcysteine, which is a direct inhibitor of ROS. These findings open new perspectives regarding the pathogenesis and therapeutic possibilities for SjS.

## Introduction

Primary Sjögren's syndrome (SjS) is a chronic autoimmune epithelitis affecting mainly women, which is characterized by lymphocytic infiltration of lachrymal and salivary glands, resulting in progressive loss of their secretory function. Patients suffer from xerophthalmia and xerostomia, also known as sicca syndrome, while systemic manifestations and multiple organ involvement are not infrequent and represent a worse clinical course of the disease, with high risk for the development of B-cell lymphoma (1–4). Although both genetic and epigenetic studies have opened new perspectives for understanding this complex disease, the etiology of SjS remains obscure (5). Histological data from salivary glands have shown activation of oxidative stress in salivary gland epithelial cells (SGECs) associated with infiltration by activated CD4(+) and CD8(+) T lymphocytes (LT) (6). Along with the progression of the disease, dendritic cells and B lymphocytes are also recruited, leading to local production of interferon (IFN) type I and type II and of circulating autoantibodies, such as anti-sicca syndrome type A (SSA/Ro) and type B (SSB/La) (7–11).

A multitude of soluble factors and cell surface molecules are upregulated in SGECs during SjS development, including both positive and negative regulators of autoimmune responses and pathologies. Among them, the intracellular adhesion molecule-1 (ICAM-1) seems to play an important role in SjS pathogenesis. The interaction of SGECs, which express ICAM-1, with immune cells through their ligands, leads to the retention of infiltrating lymphocytes. ICAM-1 upregulation in SjS was correlated not only with the lymphocytic infiltration but also with disease activity and severity (12–14). Moreover, studies in animal models have shown that blocking ICAM-1 may result in attenuation of salivary gland inflammation in early stages of the disease (15). Another example is programmed-death ligand-1 (PD-L1), a transmembrane protein, that presents a dual function. On one hand, PD-L1 is associated with epithelial cell survival and resistance to IFN-mediated apoptosis (16); on the other hand, PD-L1 downregulates various immune responses by engaging the co-inhibitory receptor programmed death-1 (PD-1) protein. When activated by its ligand, PD-1 suppresses T-cell activation and IFNγ production by TH1 cells. PD-L1 is expressed constitutively in myeloid cells and can be induced in many cell types, including epithelial cells, after exposure to pro-inflammatory stimuli. It has been shown that PD-L1 expression is elevated in SGECs of SjS patients, suggesting a potential protective role for the epithelial cells as well as an immunosuppressive role for infiltrating T cells in this disease (17, 18). As a consequence, blocking the interaction between PD-L1 and PD-1 represents an emerging therapeutic target for both autoimmune and malignant diseases (19, 20).

Reactive oxygen species (ROS) have been implicated in the induction of inflammation and tissue damage in the field of SjS (21) and other autoimmune diseases (22–25). The role of ROS in the activation of SGECs during SjS is supported by recent studies (6). First, ROS were proved to increase ICAM-1 in epithelial cell lines and to increase its ability to bind neutrophils (26), whereas treatment with N-acetylcysteine (NAC), a ROS scavenger, was able to block ICAM-1 expression in myeloid cell lines (27). ICAM-1 blockade with selective anti-ICAM-1 monoclonal antibodies prevented ROS production in epithelial cells (28). Second, the role of ROS on PD-L1 expression is also suggested as recent studies have shown an increase of PD-L1 after treatment with ROS inducers in macrophages (29). Moreover, a link between ROS and PD-L1 upregulation was described in non-small epithelial cell lung carcinoma and was correlated with cisplatin resistance (30). According to current literature, IFNs have been proven to be the main regulators of both ICAM-1 and PD-L1 expression in epithelial cells, including SGECs through the activation of the Janus kinase (JAK)/signal transducer and activator of transcription (STAT) pathway and subsequent induction of IFN gamma activated site (GAS) and IFN response element (IRE) (31–34). Our hypothesis is that part of this activation may be related to the oxidative stress.

Based on the hypothesis mentioned above and our recent study showing an interplay between ROS and the JAK/STAT pathway to control epigenetic actors (35), the aim of our study was to investigate the role of ROS on the expression of ICAM-1 and PD-L1, as well as to elucidate the potential reversible effect of JAK inhibitors and NAC in this process, a finding that could lead to the development of a novel therapeutic target in this still incurable disease.

## Materials and Methods

### Cell Culture

The human salivary gland (HSG) cell line, commonly used as an *in vitro* model to study salivary gland pathways, was used (36–39). Cells were suspended in Dulbecco's modified Eagle's medium (DMEM; Lonza Inc., Allendale, NJ), supplemented with fetal bovine serum (Eurobio, Les Ulis, France), with 2 mM L-glutamine, 250 mg/ml amphotericin B, and penicillin/streptomycin, at 37°C and with 5% CO_2_. Trypsin was used to collect cells, and cells were cultured afterward, unless otherwise specified, in the presence of 25 ng/ml type I IFNα (500 U/ml, ImmunoTools, Friesoythe, Germany); 25 ng/ml type II IFNγ (500 U/ml, ImmunoTools); 150 μM hydrogen peroxide (H_2_O_2_); 40 μM AG490, a JAK2/epidermal growth factor receptor (EGFR) inhibitor (Sigma, St. Louis, USA); 100 nM ruxolitinib, a JAK1/2 inhibitor (Jakavi, Novartis, Basel, Switzerland); and 50 mM NAC (Hidonac®, injecting solution 5 g/ml), a membrane-penetrating antioxidant and ROS modulator for 48 h.

### Quantitative PCR

For quantitative PCR, total RNA was extracted from the cells using the RNAble® (Eurobio) according to the manufacturer's instructions. The purity and quantity of the RNA were measured by determining the ratio of absorbance at 260 and 280 nm (NanoDrop® 1000, Latech). Next, 2 μg of total RNA was converted to cDNA with the Superscript II (ThermoFisher, Waltham, USA) according to the manufacturer's instructions and stored at −20°C. The master mix contained 3 μl of cDNA at dilution 1:50 (12 ng of cDNA), 1 × Power SYBR Green PCR Master Mix or 1 × Taqman assay kit (Applied Biosystems, Foster City, CA) and 250 nM of each primer. For the SYBR Green PCR: ICAM-1 (primer sense 5′-GCCGGCCAGCTTATACACAA-3′; reverse 5′-TGGCCACGTCCAGTTTCC-3′) and GAPDH (primer sense 5′-TGCCCTCAACGACCACTTT-3′; reverse 5′-GGTCCAGGGGTCTTACTCCTT-3′; For the Taqman assays: Hs00204257 (PD-L1) and Hs02758991 (GAPDH). The relative quantification of gene expression was calculated using the formula of 2-ΔΔCT with the use of GAPDH as an internal control, and results were expressed relative to the baseline.

### Flow Cytometry

For cell surface marker determination, HSG cells were cultured for 48 h in the presence of H_2_O_2_, IFNα, or IFNγ both with and without the presence of AG490, ruxolitinib, and NAC. Afterward, the effect on ICAM-1 and PD-L1 plasma membrane expression was evaluated using ICAM-1 (CD54)-FITC (Beckman Coulter, Brea, CA) and PD-L1 (CD274)-PE (Thermo Fisher Scientific, Waltham, US) anti-mouse antibodies.

For measurement of the oxidative stress, the cell-permeant 2′,7′-dichlorodihydrofluorescein diacetate (H2DCFDA, Carlsbad, CA) was used as an indicator for ROS in cells. Upon cleavage of the acetate groups by intracellular esterases and oxidation, the nonfluorescent H2DCFDA is converted to the highly fluorescent 2′,7′-dichlorofluorescein (DCF). Thus, the florescence level reflects the ROS generation in the cell. After cell collection, cells were washed with PBS, stained with 10 μg of H2DCFDA at 37°C, washed with PBS, and then fluorescence was monitored by flow cytometry. A treatment with 250 μM of H_2_O_2_ was used as a positive marker. For the evaluation of cell apoptosis, cells were recovered, washed, and stained for 15 min with FITC-conjugated annexin-V (AV)/propidium iodide (PI) according to the Beckman-Coulter apoptosis kit protocol.

### Western Blotting (WB)

A whole-protein extraction was performed by the use of a cellular lysis buffer containing 5 M sodium chloride (NaCl), 1 M Tris hydrochloride (Tris-HCl), 100 mM sodium fluoride (NaF), and 0.1 M ethylene diamine tetraacetic acid (EDTA), enriched with a cocktail of protease inhibitors. The quantity of extracted proteins was estimated with the MicroBCA Assay Protein Quantification kit (Interchim, San Diego, CA). The amounts of pSTAT1 (Y701), pSTAT3 (Y705), STAT1, and beta-actin were evaluated by WB using specific anti-mouse primary antibodies in the BD Pharmingen kit (BD Biosciences, France) and followed with the appropriate horseradish peroxidase (HRP)-conjugated secondary anti-mouse or anti-rabbit IgG antibodies according to manufacturer's instructions (anti-mouse biotinylated 1/5,000; streptavidin HRP 1/500) (GE Health Care Limited, UK). Signals were visualized by Luminata Forte Western HRP Substrate (EMD Millipore, Billerica, USA).

### Statistical Analysis

The results are expressed as arithmetic means with standard error of the mean (SEM), and differences among groups were analyzed by one-way ANOVA, and the Tukey's test was used for *post hoc* multiple comparisons test using GraphPad-Prism 7.0 software (La Jolla, CA, USA). Significance was assessed as *p* < 0.05.

## Results

### Effect of ROS on ICAM-1 and PD-L1 Expression

**Figure 1:**
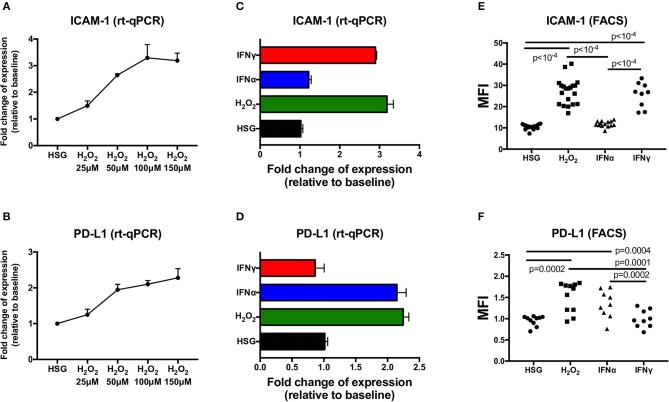
Expression of ICAM-1 and PD-L1 measured by real-time PCR and flow cytometry in the human salivary gland (HSG) cell line after 48 h of treatment with H_2_O_2_, IFNα, and IFNγ, respectively. **(A,B)** H_2_O_2_ dose effect on ICAM-1 and PD-L1 mRA expression (*n* = 3). **(C,D)** HSG (no treatment), H_2_O_2_ (150 μM), IFNα, and IFNγ effect on ICAM-1 and PD-L1 mRA expression (*n* = 3). **(E,F)** No treatment (*n* = 17 and 10, respectively), H_2_O_2_ (*n* = 20 and 11), IFNα (*n* = 14 and 9), and IFNγ (*n* = 9 both) effect on cell surface ICAM-1 and PD-L1 expression as evaluated by flow cytometry. MFI, mean fluorescence intensity; ICAM-1, intracellular adhesion molecule-1; PD-L1, programmed death ligand 1.

**Figure 2:**
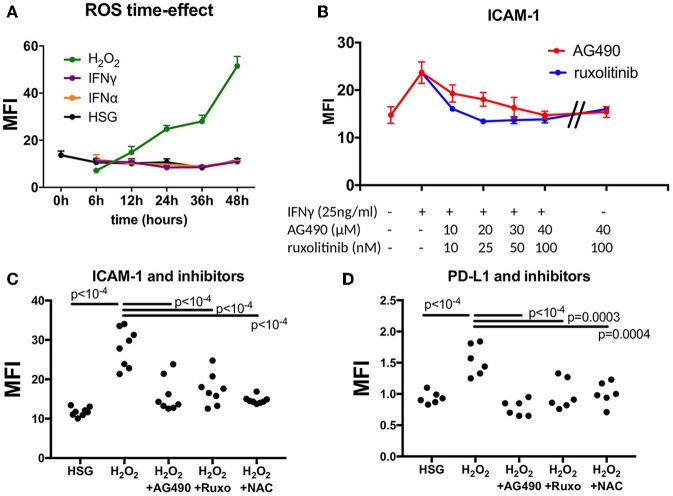
Effect of JAK inhibitors and NAC on ICAM-1 and PD-L1 induction mediated by IFNγ and H_2_O_2_. **(A)** Measurement of ROS in a time effect by flow cytometry after treatment with H_2_O_2_, IFNα, and IFNγ (*n* = 3). **(B)** Dose effect of the JAK inhibitors AG490 and ruxotinib to reverse ICAM-1 induction mediated by IFNγ. **(C)** AG490, ruxolitinib, and NAC reverse H_2_O_2_-mediated induction of ICAM-1 (*n* = 8). **(D)** AG490, ruxolitinib, and NAC reverse H_2_O_2_-mediated induction of PD-L1 (*n* = 6). ICAM-1, intracellular adhesion molecule-1; NAC, N-acetylcysteine; PD-L1, programmed death ligand 1; ROS, reactive oxygen species; H_2_O_2_, hydrogen peroxide.

To evaluate the effect of ROS mediated by H_2_O_2_ on the expression of ICAM-1 and PD-L1 compared to the effect of IFN type I (IFNα) or IFN type II (IFNγ), HSG cells were cultured for 48 h under treatment with H_2_O_2_ (25–150 μM), IFNα (25 ng/ml), or IFNγ (25 ng/ml). Then, the levels of ICAM-1 and PD-L1 were tested for transcriptional expression by real-time PCR, and for protein synthesis by flow cytometry, and mean fluorescence intensity (MFI) was reported for each treatment (Figures 1A–F). Regarding ROS activation mediated by H_2_O_2_, the expression levels of ICAM-1 and PD-L1 were increased in a dose–response relationship. At optimal concentration (150 μM H_2_O_2_), an increase for both ICAM-1 and PD-L1 was observed by real-time PCR and the induction confirmed by FACS. As expected, the positive controls IFNα and IFNγ strongly increased ICAM-1 and PD-L1, respectively. To exclude that IFNα/γ could also induce ROS production, cells were incubated for 6–48 h with IFNα, IFNγ, and H_2_O_2_, and the production of ROS was followed by flow cytometry (Figure 2A). Results revealed that only H_2_O_2_ induces ROS production in HSG cells with a maximal effect observed at 48 h, whereas no effect was observed for IFNα and IFNγ.

### JAK Inhibitors and NAC Effect on ICAM-1 and PD-L1

Next, we tested whether ICAM-1 and PD-L1 induction mediated by H_2_O_2_ was reversed by JAK inhibitors and NAC. At first, we used increasing concentrations of AG490 and ruxolitinib in the presence of IFNγ (25 ng/ml) and estimated the optimal concentration achieving ICAM-1 reduction, which was observed at 40 μM of AG490 and at 100 nM of ruxolitinib (Figure 2B). Similarly, for NAC, we used increasing concentrations of NAC in the presence of 150 μM of H_2_O_2_, and the optimal effect on ROS reduction was achieved at 50 mM (data not shown). When cells were exposed to JAK inhibitors and NAC at optimal concentrations, no effects on ICAM-1 and PD-L1 were observed. (Figure 2B and data not shown) In all conditions, apoptosis was not significantly increased (<5%) as evaluated by annexin V and propidium iodide staining (data not shown).

Afterward, cells were treated with 150 μM of H_2_O_2_ in the presence of 40 μM of AG490, 100 nM of ruxolotinib, and 50 mM of NAC for 48 h, and MFI was calculated by flow cytometry (Figures 2C,D). Results revealed a significant decrease of both ICAM-1 and PD-L1 after treatment with H_2_O_2_ in the presence of AG490, ruxolitinib, and NAC. We concluded from these experiments that ROS were effective in controlling ICAM-1 and PD-L1 expression in the HSG cell line through the JAK/STAT pathway.

### STAT1/3 Phosphorylation Status and ROS Production

**Figure 3:**
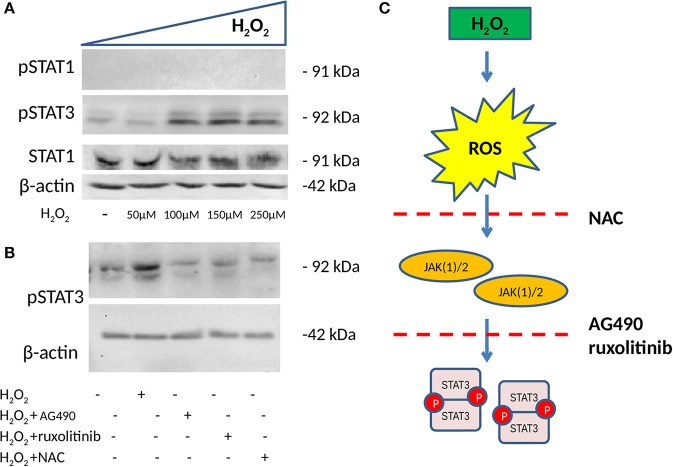
Phosphorylation status of pSTAT1 Y701 and pSTAT3 Y705 measured by WB (*n* = 3 each). **(A)** WB showing the expression of pSTAT1, pSTAT3, and total STAT1 after 48 h of treatment with increasing concentrations of H_2_O_2_. H_2_O_2_ increases pSTAT3 with the optimal effect observed at 150 μM, whereas no effect is observed on the expression of pSTAT1. **(B)** WB showing expression of pSTAT3 after 48 h of treatment with 150 μM of H_2_O_2_ with and without two JAK(1)/2 inhibitors, AG490 40 μM, a JAK2 inhibitor, ruxolitinib 100 nM a JAK1/2 inhibitor and NAC 50 mM. Both JAK1/2 inhibitors and NAC reverse H_2_O_2_-mediated induction of pSTAT3. **(C)** ROS induction mediated by H_2_O_2_ phosphorylates STAT3, a process that is reversed by NAC and JAK(1)/2 inhibitors. ROS, reactive oxygen species; H_2_O_2_, hydrogen peroxide; JAK, Janus kinase; STAT, signal transducer and activator of transcription; ICAM-1, intracellular adhesion molecule-1; PD-L1, programmed death ligand 1; WB, Western blot.

To test our hypothesis that the H_2_O_2_ effect on ICAM-1 and PD-L1 involved a JAK/STAT pathway, we further tested STAT1 (Y701) and STAT3 (Y705) phosphorylation (p) status in HSG after incubation with increasing concentrations of H_2_O_2_. Afterward, whole-cell extracts were isolated, and the pSTAT1 and pSTAT3 status was assessed by Western blot. H_2_O_2_ treatment was effective in increasing pSTAT3, with the optimal effect reported at 150 μM, whereas no effects were reported for STAT1 and pSTAT1 (Figure 3A). In addition to this, we used, as in previous experiments, the two JAK inhibitors (AG490 40 μM and ruxolitinib 100 nM) as well as NAC (50 mM) to test their effect on pSTAT3 induction mediated by H_2_O_2_. We showed that both JAK inhibitors and NAC reverse pSTAT3 activation (Figures 3B,C).

## Discussion

Our study extends the initial observations that oxidative stress and ROS are implicated in inflammation and tissue damage in SjS (6, 21, 40), as we demonstrate that ROS are effective in inducing ICAM-1 and PD-L1 expression in the HSG cell line through phosphorylation of STAT3. In addition and in agreement with *in vivo* studies supporting the involvement of JAK inhibitors and NAC in SjS (41), we have reported that the effects of H_2_O_2_ on ICAM-1 and PD-L1 can be reversed not only by JAK inhibitors but also by NAC, which is a direct ROS inhibitor. These findings open new perspectives in understanding SjS pathogenesis and the subsequent development of new therapeutic targets based on an *in vitro* cellular model using H_2_O_2_-activated HSG cells.

Studies on SGECs have shown upregulation of both ICAM-1 and PD-L1 in SjS patients (12–14, 17, 18), and until recently, their expression was related to type I and type II IFN signaling through the implication of the JAK-STAT pathway (22–24, 27, 28). However, recently published data, mainly in the field of immune cells and carcinogenesis, support the role of ROS on ICAM-1 and PD-L1 expression (26–29). In our study, we revealed a significant induction of both ICAM-1 and PD-L1 in HSG cells after treatment with H_2_O_2_ compared to the expected induction mediated by IFNs. As a consequence, part of the IFN signature described in SjS may be, in fact, related to the oxidative stress, and this is supported by a recent study using an epigenetic approach (40, 42).

In addition to this, we also demonstrated that JAK inhibitors and NAC reverse ICAM-1 and PD-L1 induction mediated by H_2_O_2_. Regarding the JAK target and based on the fact that ruxolitinib is a specific JAK1/2 inhibitor with very low affinity for non-JAK targets and that AG490 selectively inhibits JAK2 and EGFR, this supports a key role played by JAK2. Such hypothesis regarding the role of JAK2 needs confirmation with another approach. The effect of JAK inhibitors on regulating the expression of ICAM-1 and PD-L1 mediated by IFN has been studied, and it was shown that JAK inhibitors may affect the progression of autoimmune or malignant diseases by inhibiting ICAM-1 expression (43, 44) and that they could act beneficially in malignant diseases by blocking PD-L1 and reinforcing in this way the effect of antineoplastic drugs on tumor cells (16, 45, 46). Moreover, our findings are in agreement with previous reports in which the expression of ICAM-1 induced by 12-O-tetradecanoyl-phorbol-13-acetate (TPA) was linked to the generation of ROS and was reversed after treatment with NAC, a ROS scavenger (28), but also with the fact that PD-L1 was upregulated in macrophages when treated with ROS inducers (29). NAC has been proved to have a therapeutic effect in SjS mainly by attenuating ocular symptoms but is also believed to act as an anti-inflammatory agent by blocking IL-8 and ICAM-1 expression in epithelial cells, as has been described in the field of obstructive pulmonary disease (47, 48).

To investigate the potential implication of JAK/STAT pathway in ICAM-1 and PD-L1 upregulation by ROS, we investigated the phosphorylation status of pSTAT3 and not only demonstrated induction of pSTAT3 mediated by H_2_O_2_, whereas no effect was observed for pSTAT1, but also that JAK inhibitors and NAC reverse pSTAT3 induction mediated by ROS. Our findings are in agreement with previous studies where it was shown that ROS activate pSTAT3 in the field of carcinogenesis and in human T cells (25, 49) and that NAC reduces pSTAT3 expression and protein phosphorylation in animal models (50). Intracellular mechanisms linking ROS with STAT3 activation are poorly understood and may involve either intrinsic factors because the mitochondria have the capacity to interact with STAT3 or extrinsic factors through an autocrine loop with cytokines, such as IL-6 (51, 52). As a result, our findings suggest that oxidative stress mediates, through a direct or indirect mechanism, the phosphorylation of STAT3 (Y705) and the expression of ICAM-1 and PD-L1 in SGECs, a process that is reversed by JAK/STAT and ROS inhibitors.

The role of ICAM-1 and PD-L1 signaling blockade on SjS development has been described previously in animal models. Roescher et al. described that blocking ICAM-1 in non-obese diabetic mice at early stages of the disease leads to a moderate reduction of inflammation but may cause adverse events once the inflammation is established (15). In the same animal model, Zhou et al. described that upregulation of PD-L1 impedes TH1 and B-cell recruitment by reducing IFNγ production, acting in this way as a negative feedback of the disease, whereas PD-L1 blockade accelerates salivary gland dysfunction (17). However, the inhibition of PD-L1 in this manner represents a much more complex mechanism because PD-L1's functions are not restricted to a critical role in maintaining self-tolerance by blocking T-cell activation through binding PD-1 (53, 54). In autoimmune diseases, autoreactive T cells must be continually kept in check through inhibitory receptor engagement, but in SjS, strong CD8(+) activation is observed, which supports an ineffective PD-L1 pathway in such cases (55, 56). As a result, early signaling through PD-L1 would be insufficient to maintain peripheral tolerance; therefore, tissue expression of PD-L1 would be required to prevent epithelial destruction. This model explains why PD-L1 expression increases along with inflammation (55–58). On the other hand, the elevation of intracellular PD-L1 modulates epithelial cells' metabolic pathways (i) by reducing sensitivity of expressing cells to T-cell toxicity, (ii) by enhancing cell survival through an induction of AkT/mTOR pathway, and (iii) by neutralizing the proapoptotic effect of the IFN pathway (58–60). This means that co-inhibitory or co-stimulatory signals provided by PD-L1 may depend upon timing and locale, the type of cells involved, and the relative levels of PD-L1 molecules (53, 61, 62). Indeed, PD-L1 blockade in cancer cells restores their sensitivity to T-cell toxicity as well as IFN-mediated apoptotic effect (63, 64).

**Figure 4:**
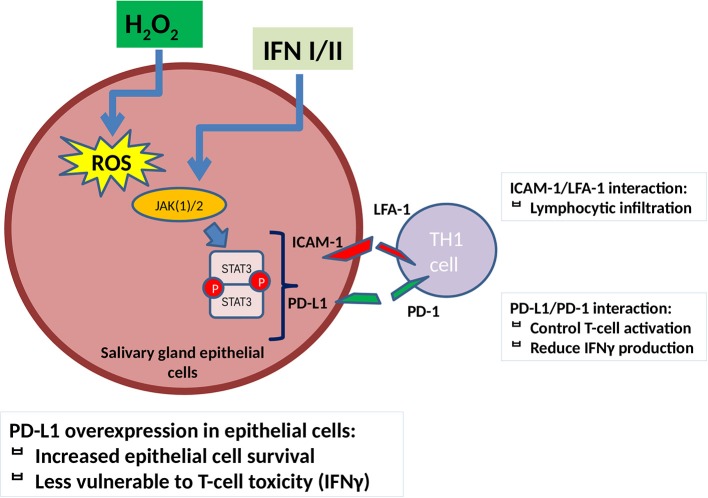
ROS contribute to ICAM-1 and PD-L1 induction in salivary gland epithelial cells through a JAK(1/)2/STAT3 pathway. ICAM-1 upregulation leads to SGEC activation and induces lymphocytic infiltration. PD-L1 reduces T-cell activation and IFNγ production on one hand but, on the other hand, contributes to SGECs activation and disease perpetuation as it increases salivary gland epithelial cell survival and reduces pro-inflammatory TH1 cell-mediated activation and toxicity. JAK(1/)2 inhibitors, AG490 and ruxolitinib, as well as NAC reverse this process as described in Figure 3. IFN(α/γ), interferon α/γ, ICAM-1, intracellular adhesion molecule-1; PD-L1, programmed death ligand 1; TH1, T helper 1 lymphocyte; ROS, reactive oxygen species; H_2_O_2_, hydrogen peroxide; TYK, tyrosine kinase; JAK, janus kinase; STAT, signal transducer and activator of transcription.

Taking into consideration the abovementioned data and combining them with our findings as described in Figure 4, we can speculate that oxidative stress in salivary glands of patients with SjS leads to an upregulation of ICAM-1 and PD-L1 through the implication of a ROS/JAK(1/)2/STAT3 pathway, which acts synergistically with IFN/JAK/STAT-mediated effect on ICAM-1 and PD-L1 expression, initiating in this manner the adhesion and infiltration of activated T cells. PD-L1 is activated along with initiation of inflammation as a negative feedback, but during the development of the disease, there is an inability to control tissue damage and on the contrary contributes to SGEC activation and increased survival and subsequently to disease perpetuation. The administration of JAK inhibitors and NAC reverses ICAM-1 and PD-L1 induction mediated by ROS and strengthens their ability to reduce ICAM-1 and PD-L1 expression mediated by IFNs, a fact that could act beneficially if administered early in disease development.

## Conclusion

Adhesion molecules and cell surface immunomodulatory factors are suspected of having an important role in SjS pathogenesis. In our study, we demonstrated in the HSG cell line that ROS induce expression of ICAM-1 and PD-L1 through activation of STAT3. JAK inhibitors and NAC reverse this process, reducing ICAM-1 and PD-L1 expression mediated by ROS. The effect of JAK inhibitors and NAC on ICAM-1 and PD-L1 production could act beneficially in SjS, reducing activation of SGECs and subsequently reducing inflammation and lymphocytic infiltration.

## Data Availability Statement

The datasets generated for this study are available on request to the corresponding author.

## Author Contributions

AC, AB, and YR designed the study. AC, PA, and CL performed the research. AC, PA, GD, KZ, AB, and YR analyzed the data. YR and PA prepared the initial draft. The final manuscript was read and approved by all authors.

### Conflict of Interest

The authors declare that the research was conducted in the absence of any commercial or financial relationships that could be construed as a potential conflict of interest.

## Abbreviations

SjS: primary Sjögren's syndrome
ROS: reactive oxygen species
IFNα: interferon α
IFNγ: interferon γ
SGECs: salivary gland epithelial cells
LT: T-lymphocytes
ICAM-1: intracellular adhesion molecule-1
PD-L1: programmed death ligand-1
JAK: Janus kinase
STAT: signal transducer and activator of transcription
WB: Western blotting
PD-1: programmed death-1
GAS element: IFN-gamma activated site
MFI: mean fluorescence intensity.
